# Fully automated radiosynthesis of [^68^Ga]Ga-FAPI-46 with cyclotron produced gallium

**DOI:** 10.1186/s41181-023-00216-0

**Published:** 2023-10-16

**Authors:** Adam J. Rosenberg, Yiu-Yin Cheung, Fei Liu, Carina Sollert, Todd E. Peterson, Jonathan A. Kropski

**Affiliations:** 1 Vanderbilt University Institute of Imaging Science, Vanderbilt University Medical Center, Nashville, TN 37232 USA; 2 Department of Radiology and Radiological Sciences, Vanderbilt University Medical Center, Nashville, TN 37232 USA; 3 Vanderbilt Ingram Cancer Center, Nashville, TN 37232 USA; 4 GE HealthCare, Uppsala, Sweden; 5 Division of Allergy, Pulmonary, and Critical Care Medicine, Department of Medicine, Vanderbilt University Medical Center, Nashville, TN 37232 USA

**Keywords:** FAPI, Radiolabeling, Gallium-68, Cyclotron, Automation, GMP compliance, Radiochemistry, Radiopharmaceuticals

## Abstract

**Background:**

Radiopharmaceuticals capable of targeting the fibroblast activation protein have become widely utilized in the research realm as well as show great promise to be commercialized; with [^68^Ga]Ga-FAPI-46 being one of the most widely utilized. Until now the synthesis has relied on generator-produced gallium-68. Here we present a developed method to utilize liquid-target cyclotron-produced gallium-68 to prepare [^68^Ga]Ga-FAPI-46.

**Results:**

A fully-automated manufacturing process for [^68^Ga]Ga-FAPI-46 was developed starting with the ^68^Zn[p,n]^68^Ga cyclotron bombardment to provide [^68^Ga]GaCl_3_, automated purification of the [^68^Ga]GaCl_3_, chelation with the precursor, and final formulation/purification. The activity levels produced were sufficient for multiple clinical research doses, and the final product met all release criteria. Furthermore, the process consistently provides < 2% of Ga-66 and Ga-67 at the 4-h expiry, meeting the Ph. Eur. standards.

**Conclusions:**

The automated radiosynthesis on the GE FASTlab 2 module purifies the cyclotron output into [^68^Ga]GaCl_3_, performs the labeling, formulates the product, and sterilizes the product while transferring to the final vial. Production of > 40 mCi (> 1480 MBq) of [^68^Ga]Ga-FAPI-46 in excellent radiochemical yield was achieved with all batches meeting release criteria.

## Background

PET imaging with fibroblast activation protein inhibitors (FAPI) has attracted great interest throughout the imaging community due to FAP’s overexpression in many cancers and inflammatory diseases, and low expression in the non-target tissue; giving it an excellent contrast ratio (Loktev et al. 2018; Glatting et al. 2022; Wang et al. 2022; Chen et al. 2020; Gilardi et al. 2022). FAPI imaging has the potential to replace much of the current usage of [^18^F]FDG due to its higher specificity, sensitivity, and potential to be part of a theranostic pair (Calais and Mona 2020).

[^68^Ga]Ga-FAPI-46 ([^68^Ga]FAPI-46) is a small molecule that binds specifically to FAP, which is highly expressed in tumor-associated fibroblasts but not in normal tissues, allowing for high contrast imaging of cancerous tissues. [^68^Ga]FAPI-46 has been used across cancer types, including lung (Wang et al. 2022; Borgonje et al. 2022), head/neck (Promteangtrong et al. 2022), colorectal (Mona et al. 2022), urothelial (Unterrainer et al. 2022), breast (Backhaus et al. 2022), ovarian (Siripongsatian et al. 2022), and numerous other types (Gilardi et al. 2022; Mona et al. 2022; Kratochwil et al. 2019). As a gallium-68 labeled radiopharmaceutical, [^68^Ga]FAPI-46 has been prepared using a ^68^Ge/^68^Ga generator either manually (Loktev et al. 2018, 2019; Meyer et al. 2020) or via automated synthesis modules (Spreckelmeyer et al. 2020; Da Pieve et al. 2022; Alfteimi et al. 2022; Boonkawin and Chotipanich 2021). While use of generators for gallium-68 has a long history, they remain limited for use in dedicated research facilities by their high fixed-cost unrelated to the level of usage. Without a high level of clinical usage, scattered and unpredictable basic and clinical research needs lead to unsustainable capital costs to ensure generator access. Furthermore, they require replacement every 6–9 months as well as have diminishing activity levels.

The cyclotron-based route via liquid targets (Alves et al. 2017; Pandey et al. 2019; Rodnick et al. 2020, 2021) can produce gallium-68 on-demand with workflows similar to fluoride-18 production. The target matrix consists of enriched zinc-68 in an aqueous nitric acid solution. Around 3–4 GBq unprocessed ^68^Ga at the end of a 60 min irradiation can be produced routinely. In order to make use of the ^68^Ga for tracer labelling, chemical processing of the irradiated target material to convert the crude ^68^Ga to [^68^Ga]GaCl3 and to remove zinc and other metals is required. After labelling and final purification of the tracer typical activity yields are around 2 GBq (Rodnick et al. 2020).

The FDA approval of the two tracers [^68^Ga]Ga-DOTA-TOC and [^68^Ga]Ga-PSMA-11 (Hennrich and Eder 2021; Carlucci et al. 2021; Hennrich and Benešová 2020) and the update of the Ph. Eur. for accelerator-produced ^68^Ga highlights the increasing demand and relevance of cyclotron-produced 68Ga using the liquid target route (Gallium (68Ga) Chloride (accelerator produced) solution for radiolabelling 2020).

The production of ^68^Ga with solid ^68^Zn targets (Thisgaard et al. 2021; Becker et al. 2021) answers an increasing demand for higher yields and larger number of patient doses per batch. Efforts to simplify and optimize solid target workflows and processes have been made (Svedjehed et al. 2022), but in comparison to liquid target more specialized infrastructure and laboratory space are required. For research or a smaller clinical demand, liquid target offers good coverage.

In order to allow access to [^68^Ga]FAPI-46 for our investigators we developed its automated radiosynthesis using cyclotron-produced gallium-68. The process performs the purification of the gallium-68 target solution, radiolabeling, and purification all on a single cassette with no manual manipulations required.

## Results

**Scheme 1:**
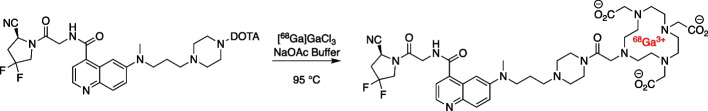
Synthesis of [^68^Ga]Ga-FAPI-46

The radiosynthesis of [^68^Ga]Ga-FAPI-46 is shown in Scheme 1. Our approach was based upon the previously published reports (Spreckelmeyer et al. 2020; Rodnick et al. 2020). Previously, [^68^Ga]FAPI-46 has been prepared using ^68^Ge/^68^Ga generators using either a manual or automated process. However, to date, there has been no reported process for making use of cyclotron-produced gallium-68 to prepare [^68^Ga]FAPI-46. We elected to make use of the GE FASTlab 2 for both the purification of the [^68^Ga]GaCl_3_ and for its subsequent use in the radiolabeling. In addition to commercially available cassettes and kits, the FASTlab has a basic cassette skeleton and components available for customized design and radiosynthesis implementation. We were able to make use of this to customize the cassette layout and accomplish the production of [^68^Ga]FAPI-46. While starting with the previously developed cassette layouts of [^68^Ga]GaCl_3_ and [^68^Ga]PSMA-11 already developed on the FASTlab, there was no straightforward method to perform a trap and release purification on the cassette while also not requiring a final vial already charged with formulation solution due the limited positions on the cassette skeleton. It was decided to mimic the manual radiosynthesis used by Sofie as well as other automated syntheses of [^68^Ga]FAPI-46, which did not make use of a final SPE purification (Spreckelmeyer et al. 2020; Private communications from Sofie). In particular, the use of a CM cartridge to remove free-gallium was utilized, as this did not require additional skeleton positions (Spreckelmeyer et al. 2020). Using the optimized conditions established, 43.7 ± 2.5 (n = 3) mCi (1617 ± 92.5 MBq) of [^68^Ga]FAPI-46 was produced. This is similar to the yields for [^68^Ga]PSMA-11 previously reported (Rodnick et al. 2020).

The production of [^68^Ga]GaCl_3_ has been previously demonstrated using a liquid cyclotron target and the FASTlab 2 module to produce roughly 50 mCi (1850 MBq) after purification (Rodnick et al. 2020). Briefly, the [^68^Ga]GaCl_3_ is obtained by purifying the proton-bombarded [^68^Zn]Zn(NO_3_)_2_ solution delivered from the cyclotron to an external activity receiving vial. The purification process is based on a three-column purification. The target solution is diluted to ≤ 0.1 M nitric acid by addition of water. The ^68^Ga is trapped on the first column (ZR Resin) while the zinc is not trapped on the column under these conditions. Residual zinc is then washed off the column with ~ 0.1 M nitric acid. Next, the ^68^Ga is eluted with ~ 1.75 M hydrochloric acid (HCl), passed through the anion exchange cartridge, and trapped in the 3rd column (TK 200 Resin). This resin is washed with mildly acidic (~ 0.1 M HCl) 2 M sodium chloride solution and finally eluted into the reactor, to which the DOTA-FAPI-46 precursor had been added, with water followed by dilute HCl. Labelling of DOTA-FAPI-46 in an acetate buffer takes place at 95 °C over 10 min. The reaction mixture is then diluted with 0.9% saline and passed through the Accell CM cartridge to the final vial through a 0.22-micron sterilizing filter. The reactor is rinsed with additional 0.9% saline, which is passed through the 0.22-micron sterilizing filter directly into the final vial. The radiosynthesis required approximately 45 min from the end of bombardment (EOB) to the drug product solution being deposited in the final vial, depending on the user interactions (Table 1).

**Table 1:** Radiosynthesis results

Quality control test	Specifications	Qual Run 1	Qual Run 2	Qual Run 3
Appearance	Clear, colorless, no particulates	Clear, colorless, no particulates	Clear, colorless, no particulates	Clear, colorless, no particulates
Filter integrity	Pass	Pass	Pass	Pass
pH	4.0–7.0	4.5	4.5	4.5
Radiochemical purity (%) (HPLC)	NLT 90% *	95.31	95.24	96.40
Radiochemical purity (%) (iTLC)	NLT 90%	> 99	> 99	> 99
Radionuclidic identity (t1/2, minutes)	64–67	64.46	67.72	69.65
Bacterial endotoxin levels (EU/mL)	< 8.75 EU/mL	< 1.00 EU/mL	< 1.00 EU/mL	< 1.00 EU/mL
Sterility (observed growth after 14 d)	No growth	No growth	No growth	No growth
Radiochemical identity (% difference from reference Std) by HPLC	NMT 10% *	1.46%	1.55%	1.30%
Amount of product @ EOS	N/A	45.8 mCi (1695 MBq)	41.1 mCi (1521 MBq)	44.2 mCi (1635 MBq)
Gallium-66 and Gallium-67 impurities @ EOS	< 2.0%*	0.132%	0.145%	0.159%

*NLT* not less than, *NMT* not more than, *EOS* end of synthesis

^*^Periodic quality indicator test

**Fig. 1:**
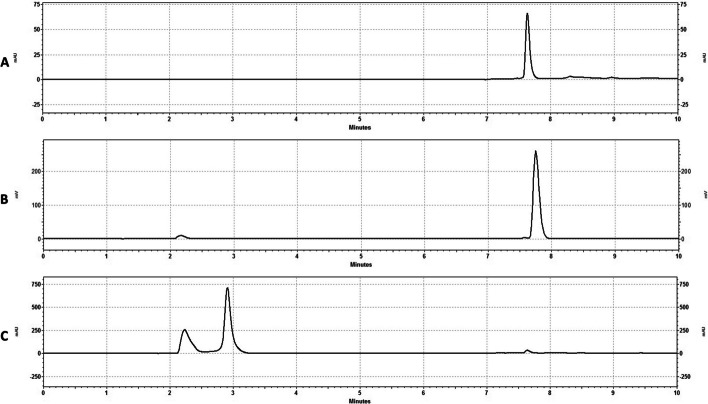
HPLC Chromatograms of reference standard and [^68^Ga]FAPI-46. **A** UV absorbance (254 nm) of Ga-FAPI-46 reference standard. **B** Radiochromatogram of [^68^Ga]FAPI-46. **C** UV absorbance (254 nm) of [^68^Ga]FAPI-46 corresponding to B

As shown in Fig. 1 the retention time of the Ga-FAPI-46 reference standard material was 7–8 min, and the relative retention time of the labeled [^68^Ga]FAPI-46 was within a 1% difference. Furthermore, TLC analysis confirms minimal free gallium.

As with many gallium radiosyntheses it is critical to minimize any potential sources of metal ion contaminants. All reagents used were of the highest quality available and manipulations were carried out using metal-free materials to the greatest possible extent; however, a designated metal free preparation area was not required.

**Fig. 2:**
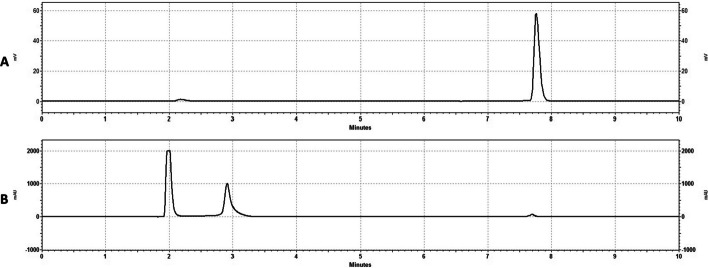
HPLC Chromatograms at 4h Expiry. **A** Gamma Trace of [^68^Ga]FAPI-46 at expiry (240 min). **B** UV trace of [^68^Ga]FAPI-46 corresponding to (**A**)

Stability studies conducted at room temperature showed no degradation of the product out to 4-h, with consistent radiochemical purities by HPLC shown; and importantly no increase in free-gallium (Table 2). The pH of the solution was also consistent demonstrating no radiolytic induced pH change. Representative chromatograms of the [^68^Ga]FAPI-46 at the 4-h expiry are shown in Fig. 2.

**Table 2:** Stability of [^68^Ga]FAPI-46

Batch	Elapsed time after synthesis (min)									
	0 min (EOS)		90 min		150 min		210 min		240 min	
	RCP (%)	pH	RCP (%)	pH	RCP (%)	pH	RCP (%)	pH	RCP (%)	pH
Q1	95.31	4.5	96.76	4.5	96.76	4.5	96.76	4.5	96.76	4.5
Q2	95.24	4.5	95.78	4.5	95.78	4.5	95.78	4.5	95.78	4.5
Q3	96.40	4.5	97.51	4.5	97.51	4.5	97.51	4.5	97.51	4.5

*RCP* radiochemical purity by HPLC

## Discussion

Using developed cyclotron-production and purification methods, gallium-68 chloride was prepared on an automated cassette for use in radiolabeling FAPI-46. The labeling, purification, and formulation steps were accomplished using the same cassette on a broadly installed radiochemistry module (GE FASTlab 2). Previously, [^68^Ga]FAPI-46 has been prepared using generator-produced gallium-68 by both automated and manual methods. This approach was considered for use in our facility, but due to the cost and availability difficulties associated with a gallium generator we elected to pursue an on-demand cyclotron-based approach. Our facility only performs limited gallium-68 work on a difficult to predict basis, so we are not able to support consistent gallium generator purchases. While it is also possible to use the cyclotron-produced method to prepare gallium-68 chloride and then use it in either a manual or automated approach separately, this was not pursued due to the advantages of having a single cassette for both the gallium-68 purification and radiosynthesis. The workflow is very similar to fluorine-18 operations, and has been easy to incorporate into our manufacturing operations.

Using the previously developed method for purification of the cyclotron-produced gallium-68, a successful GMP product validation campaign was carried out with all three runs meeting acceptance criteria suitable for clinical use; and giving > 40 mCi (1480 MBq) of [^68^Ga]FAPI-46 (Table 1). Critically, this method of production gives very low Ga-66 and Ga-67 impurities (Table 1). This radiosynthesis provides [^68^Ga]FAPI-46 in high radiochemical purity (> 95%) and in a fully GMP-compliant fashion. The resulting [^68^Ga]FAPI-46 is stable out to its 4-h expiry, with no observed radioimpurity formation or increase in free gallium-68 (Fig. 2, Table 2).

## Conclusions

We have successfully developed an automated radiosynthesis of [^68^Ga]FAPI-46 using cyclotron-produced gallium on the commercially available GE FASTlab 2 platform. Use of this method allows for a simple, reliable process that gives good radiochemical yields and an excellent purity profile. The [^68^Ga]FAPI-46 produced passes all release criteria as well as reliably providing a sterile and pyrogen-free GMP-compliant final product.

## Methods

### General

All chemicals were obtained from commercial sources and were of analytical, ACS, Trace-metal, Ultrapur, or USP quality (Millipore-Sigma, USA) and were used without further purification. All preparations were performed under metal-free conditions, with single-use plastic consumables. Separation cartridges were obtained from commercial sources and were of the highest quality available (Triskem, France; Waters, USA). FASTlab developer kits, including cassette skeletons, vials, and tubing were obtained from GE Healthcare (Waukesha, WI, USA). FAPI-46 precursors and Ga-FAPI-46 reference standard were provided by Sofie Biosciences (Dulles, VA, USA). All GMP operations, including in-process material preparation and cassette assembly, are recorded in an electronic laboratory information management system and linked to the operator; in a manner consistent with USP <823>. All materials are traceable to the raw material components and are accepted under standard GMP inventory procedures.

### Preparation of in-process materials

#### 4 M aqueous hydrochloric acid

To a sterile 50 mL centrifuge tube 15–20 mL of Ultrapur water was added, followed by careful addition of 16 mL 30% Ultrapur Hydrochloric Acid (HCl). The resulting solution was diluted to 40 mL by the addition of Ultrapur water and vortexed to mix. The resulting solution was dispensed into 13 mm FASTlab vials in 4 mL aliquots.

#### Sodium acetate buffer (pH 4.8)

To a sterile 50 mL sterile centrifuge tube was added 2.46 g of Sodium Acetate (NaOAc), followed by 30 mL of Ultrapur water. The pH was adjusted to 4.8 by dropwise addition of 4 M HCl (prepared above).

#### 0.6 M aqueous nitric acid

In a 50 mL sterile centrifuge tube 1.5 mL of Nitric Acid (trace metal grade) and 38.5 mL of Ultrapur water were mixed. The resulting solution was dispensed into 13 mm FASTlab vials in 4 mL aliquots.

#### 3 M aqueous sodium chloride

To a sterile 50 mL sterile centrifuge tube was added 7.02 g of Sodium Chloride, followed by 20 mL of Ultrapur water. The solution was vortexed to ensure dissolution of the sodium chloride, diluted to 40 mL by addition of Ultrapur water, and again vortexed to mix the solution completely. The resulting solution was dispensed into 13 mm FASTlab vials in 4 mL aliquots.

### Cyclotron production of gallium-68

Gallium-68 was obtained in the chemical form of [^68^Ga]gallium(III) nitrate ([^68^Ga]Ga(NO_3_)_3_) via the ^68^Zn(*p*,n)^68^Ga nuclear reaction, by irradiating a cyclotron liquid target containing 1 M [^68^Zn]Zn(NO_3_)_2_ in dilute nitric acid with a proton beam of 30–40 μA for 60 min (PETtrace cyclotron, GE, Uppsala, Sweden). The target media was prepared from isotopically enriched [^68^Zn]ZnO (Isoflex, USA) with the addition of Ultrapur water and 70% nitric acid (trace metal grade). After the production, a cleaning bombardment was performed on dilute nitric acid at 30–40 μA for 20–60 min, followed by emptying of the target to a waste container and drying the target.

#### Automated radiosynthesis of [^68^Ga]Ga-FAPI-46

**Fig. 3:**
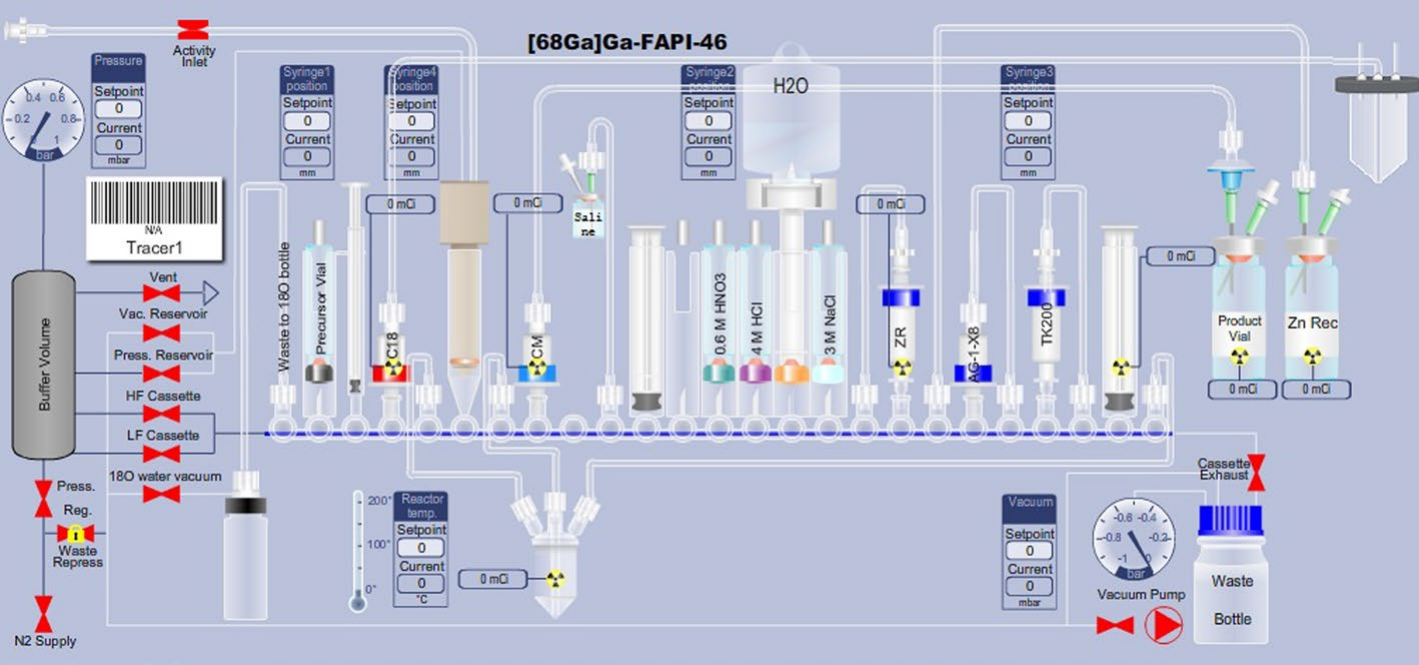
FASTlab Cassette Schematic as shown in the FASTlab multitracer software

For the synthesis of [^68^Ga]Ga-FAPI-46 using cyclotron-produced ^68^Ga, previously known conditions were adapted (Spreckelmeyer et al. 2020; Rodnick et al. 2020). The GE FASTlab 2 automated radiosynthesis module was used with the developer control software. The cassettes were assembled in-house using Developer kit components as shown in Fig. 3 and the positions listed in Table 3.

**Table 3:** Table of cassette design with amounts of reagents

Position	Description	Parameter
1	14 cm transfer line to the O-18 waste bottle	–
2	Precursor vial (11 mm)	50 μg FAPI-46, 3 mg ascorbic acid, 1.4 mL sodium acetate buffer
3	1 mL syringe	–
4	C18 light Sep-Pak with 42 cm transfer line to the central line of the activity receiving vial	Activity receiving vial is connected to the cyclotron transfer line via and to the FASTlab activity inlet line
5	14 cm transfer line to reactor	–
6	Conical reservoir (not used)	–
7	14 cm transfer line to reactor	–
8	Accell plus CM plus short cartridge with 42 cm transfer line to final filter and final vial	–
9	Empty	–
10	42 cm transfer line to 0.9% saline	50 mL vial of 0.9% saline with spinal needle and vent needle
11	6 mL syringe	–
12	Empty	–
13	Nitric acid vial (13 mm)	4 mL 0.6 M HNO_3(aq)_
14	Hydrochloric acid (13 mm)	4 mL 4 M HCl_(aq)_
15	Sterile water for injection bag	Water spike with 100 mL SWFI bag
16	Sodium chloride	4 mL 3 M NaCl_(aq)_
17	14 cm transfer line to ZR-Resin	–
18	ZR resin cartridge (2 mL)	–
19	42 cm transfer line to zinc waste vial	–
20	Anion exchange cartridge	Ag1-X8 resin cartridge, 1 mL resin
21	14 cm transfer line to anion exchange cartridge	–
22	TK200 resin cartridge (2 mL)	–
23	14 cm transfer line to TK200 Cartridge	–
24	6 mL syringe	–
25	42 transfer line to reactor	–

#### Cassette testing and preliminary steps

Prior to activity transfer to the FASTlab, the cassette is tested for leaks/blockages and the system is made ready for the synthesis. The FASTlab automatically tests the cassette skeleton integrity for leaks and blockages, as well as all flow paths other than from the activity receiving vial and the final product output line. Once the testing is completed the FASTlab will also condition the cartridges as required and prepare the system for the synthesis.

#### Automatic radiosynthesis using the GE FASTlab 2 module

At the end of bombardment (EOB) gallium-68 was transferred from the cyclotron to the activity receiving vial attached to the cassette. The solution was diluted and the gallium-68 purified according to established procedures on the FASTlab (Rodnick et al. 2020), with the addition of an anion exchange cartridge. The resulting [^68^Ga]GaCl_3_ was transferred to the reactor, where the precursor solution (50 μg dissolved in 1.4 mL of sodium acetate buffer (pH = 4.8) with 3 mg ascorbic acid) had already been transferred. The resulting reaction solution was heated to 95 °C for 10 min, cooled to room temperature and diluted with 0.9% saline. The resulting solution is passed through a CM cartridge directly to the final vial through a 0.22-micron sterilizing filter. The reactor is rinsed with additional 0.9% saline which is passed through the 0.22-micron sterilizing filter directly into the final vial (Pall, USA, Part#: AEF1NTE) to provide the [^68^Ga]Ga-FAPI-46 as a ready to inject solution. The radiosynthesis required approximately 45 min from the end of bombardment (EOB) to the drug product solution being deposited in the final vial. A more detailed description of the method can be found in Table 4.

**Table 4:** Radiosynthetic steps in the synthesis of [^68^Ga]FAPI-46

Step	Description	Parameter
1	Transfer of precursor to reactor and bombardment	1.1 mL precursor in 1M NaOAc buffer to reactor
2	Delivery of [^68^Ga]Ga(NO_3_)_2_ to receiving vial	–
3	Dilute of [^68^Ga]Ga(NO_3_)_2_ solution to < 0.1 M HNO_3_	6 mL H_2_O
4	Trapping on ZR resin	
5	Washing of ZR resin	2 × 7.3 mL 0.1 M HNO_3_
6	Elution of ZR resin and trapping on TK resin	5–6 mL ~ 1.75 M HCl
7	Washing of TK resin	3.5 mL 2.0 M NaCl in 0.13 M HCl
8	Elution of TK resin to reactor	2.7 mL H_2_O and dilute HCl
9	Radiolabeling	95 °C for 10 min
10	Cooling and dilution	2 mL 0.9% Saline
11	Transfer to final vial	Passing through CM cartridge and sterilization filter
12	Reactor wash	4 mL 0.9% saline
13	Transfer to final vial	Passing through CM cartridge and sterilization filter

#### Quality control for [^68^Ga]Ga-FAPI-46

Quality control results from each of the three qualification batches are shown in Table 1. All quality control measurements were performed on GMP-qualified instruments unless otherwise stated. After completion of the synthesis an aliquot of the product was withdrawn for quality control, determining the appearance by visual inspection and radionuclidic identity by half-life measurement using a dose calibrator (Capintec CRC-15 Dual PET). Radiochemical and chemical purities were analyzed by analytical HPLC using a Phenomenex Gemini C18 column (150 × 4.6 mm) with a guard column (Phenomenex SecurityGuard C18, 4 × 3 mm) [^68^Ga]Ga-FAPI-46 RT = 7.7 min (0–100% MeCN (/w 0.1% TFA) in H2O (/w 0.1% TFA) 1 mL/min, 254 nm). Radiochemical purity was also determined by iTLC (75% MeOH in 5M Ammonium Acetate_(aq)_ used as eluent). Apyrogenicity tests were performed in-house using Endosafe-Nextgen PTS (Charles River Laboratories Inc.) to ensure that doses of [^68^Ga]Ga-FAPI-46 contained < 8.75 endotoxin units (EU) per mL. ColorpHast® pH indicator strips (EMD Chemicals Inc.) were used to determine pH of the final product. Final filter integrity testing was performed on the sterilizing filter by standard bubble-point. Sterility testing was performed via direct inoculation into growth media. Stability testing of the [^68^Ga]Ga-FAPI-46 product was performed at periodic times over four hours, testing for radiochemical purity by HPLC and pH. Gallium-66 and Gallium-67 impurities present in the final product are determined though measurement of the residual drug product per USP <821> using a GeGI (PHDs) high-purity germanium detector. Gallium-66 is quantified by use of the 833 keV and 1039 keV emissions, and Gallium-67 is quantified by use of the 300 keV and 393 keV emissions; the resulting activities are decay-corrected to EOS. The results of the three consecutive qualification runs are shown in Table 1.

## Data Availability

All data generated or analyzed during this study are included in this published article [and its supplementary information files].
